# A New Method of Recording the Motions of the Soft Palate

**Published:** 1884-12

**Authors:** 


A NEW METHOD OF RECORDING THE MOTIONS OF THE SOFT PALATE. By Harrison Allen, M. D.—This volume consists of extracts from the transactions of the College of Physicians of Philadelphia, and describes a new method of recording the motions of the soft palate, with numerous illustrations of the text. Its perusal cannot fail to instruct the student, both medical and dental, on a complex subject and its author deserves great credit for his investigations in such a direction. Publishers: P. Blakiston, Son & Co., Philadelphia.

